# Patient-Derived Primary Cancer-Associated Fibroblasts Mediate Resistance to Anti-Angiogenic Drug in Ovarian Cancers

**DOI:** 10.3390/biomedicines11010112

**Published:** 2023-01-01

**Authors:** Raed Sulaiman, Pradip De, Jennifer C. Aske, Xiaoqian Lin, Adam Dale, Nischal Koirala, Kris Gaster, Luis Rojas Espaillat, David Starks, Nandini Dey

**Affiliations:** 1 Department of Pathology, Avera Cancer Institute, Sioux Falls, SD 57105, USA; 2 Translational Oncology Laboratory, Avera Research Institute, Sioux Falls, SD 57105, USA; 3 Department of Internal Medicine, University of South Dakota SSOM, USD, Sioux Falls, SD 57105, USA; 4 Assistant VP Outpatient Cancer Clinics, Avera Cancer Institute, Sioux Falls, SD 57105, USA; 5 Department of Gynecologic Oncology, Avera Cancer Institute, Sioux Falls, SD 57105, USA

**Keywords:** angiogenesis, patient-derived CAFs, endothelial cells, hybrid co-culture, lenvatinib, cord formation assay, ovarian cancers, anti-angiogenic drug resistance

## Abstract

Ovarian cancers rank first in both aggressiveness and dismal prognosis among gynecological neoplasms. The poor outcome is explained by the fact that most patients present with late-stage disease and progress through the first line of treatment. Ovarian neoplasms, especially epithelial ovarian cancers, are diagnosed at advanced/metastatic stages, often with a high angiogenesis index, one of the hallmarks of ovarian cancers with rapid progression and poor outcome as resistance to anti-angiogenic therapy develops. Despite therapy, the metastatic progression of aggressive ovarian cancer is a spectacularly selective function of tumor cells aided and abetted by the immune, mesenchymal and angiogenic components of the tumor microenvironment (TME) that enforces several pro-metastatic event(s) via direct and indirect interactions with stromal immune cells, cancer-associated fibroblasts (CAFs), and vascular endothelial cells. Since transdifferentiation of tumor endothelium is one of the major sources of CAFs, we hypothesized that ovarian CAF plays a critical role in resisting anti-angiogenic effects via direct crosstalk with endothelium and hence plays a direct role in the development of resistance to anti-angiogenic drugs. To test the hypothesis, we set up a hybrid ex vivo model for co-culture comprising Patient-Derived ex vivo primary CAFs from ovarian tumor samples and human umbilical vein endothelial cells (HUVEC). Patient-Derived CAFs were characterized by the mRNA and protein expression of positive (SMA, S100A4, TE-7, FAP-A, CD90/THY1), negative (EpCAM, CK 8,18, CD31, CD44, CD45), functional (PDGFRA, TGFB1, TGFB2, TGFRA) and immunological markers (PD-L1, PD-L2, PD-1) associated with CAFs by qRT-PCR, flow cytometry, Western blot, and ICC. Data from our HUVEC-on-CAF ex vivo Hybrid Co-Culture (HyCC) study demonstrate the pro-angiogenic effect of Patient-Derived ovarian CAFs by virtue of their ability to resist the effect of anti-angiogenic drugs, thereby aiding the development of resistance to anti-angiogenic drugs. Ascertaining direct experimental proof of the role of CAFs in developing resistance to specific anti-angiogenic drugs will provide an opportunity to investigate new drugs for counteracting CAF resistance and "normalizing/re-educating" TME in aggressive ovarian cancers. Our data provide a unique experimental tool for the personalized testing of anti-angiogenic drugs, positively predicting the development of future resistance to anti-angiogenic drugs well before it is clinically encountered in patients.

## 1. Introduction

Angiogenesis, the formation of new vessels from pre-existing vasculature, is one of the hallmarks of ovarian neoplasms and a phenotype essential for the progression of the disease [1,2]. During the progression of the disease, tumor cells signal to coerce support from the tumor microenvironment (TME). Metastatic progression of a solid tumor, irrespective of therapy, is aided and abetted by all three components of the TME. The metastatic event(s) associated with the progression within a tumor is a stupendously selective function of tumor cells in the context of stromal cells of their TME [3,4]. The process is mediated via complex and dynamic crosstalk not only by direct and indirect interaction with tumor cells but by a direct interaction among the components of the tumor-TME, immune cells, cancer-associated stromal fibroblasts (CAFs), and vascular endothelial cells.

Ovarian neoplasms account for a fifth of cancer deaths in women, causing more deaths than any other cancer of the reproductive system (American Cancer Society statistics 2022). According to the estimates of the American Cancer Society for ovarian cancers for 2022, (1) close to 20K women will get newly diagnosed, and (2) over 12K patients will die from ovarian cancer. [5] With 75% of patients presenting stage III or IV disease, especially epithelial ovarian cancers [6,7], ovarian cancers are the most aggressive gynecological neoplasms with a dismal prognosis. Although more than 80% (out of 75% of patients who present with stage III or IV disease) of these patients respond and benefit from first-line therapy, recurrence is common at a median of 15 months from diagnosis. Second-line treatments are also not curative [8]. Approximately 80% of ovarian cancers are epithelial ovarian cancer (EOC) [8,9], diagnosed at advanced/metastatic stages [10], and present a high angiogenesis index that facilitates rapid tumor progression [1,11]. Vascular endothelial growth factors (VEGF) secreted by ovarian tumor cells promote angiogenesis via its receptors on endothelial cells and directly correlate with poor prognosis indicating a role of VEGF signaling in disease progression [12,13].

Combination vascular targeted therapy is a recognized promising approach for patients with platinum-resistant recurrent ovarian cancers [14]. Three categories of anti-angiogenic agents include agents targeting the VEGF/VEGFR pathway, receptor tyrosine kinase, and non-VEGF/VEGFR targets. Bevacizumab is historically the first targeted anti-angiogenic agent against the VEGF/VEGFR pathway approved by the FDA in ovarian cancer and is widely studied to be implemented in the first-line treatment [2,7,15,16]; with platinum-based chemotherapy in platinum-sensitive (OCEANS trial) [17] and platinum-resistant (AURELIA trial) [18] recurrent ovarian cancers. However, bevacizumab is efficacious only in a limited set of patients [19], and several proposed resistance mechanisms have been suggested [20,21]. Phase III trials of bevacizumab demonstrated improved PFS (progression-free survival) in patients with recurrent ovarian cancers, irrespective of the sensitivity of platinum, but bevacizumab failed to improve OS (overall survival) in ovarian cancer patients [20]. Literature suggests that the resistance to anti-angiogenic therapy is multi-factorial [22].

CAFs comprise the bulk of TME and share the most versatility in origin and function, making CAF clinically and therapeutically relevant [23,24,25,26,27]). The role of stromal CAF in cancer is undeniable in the production of growth factors, chemokines, and extracellular matrix-mediated angiogenic recruitment of endothelial cells and pericytes [28,29]. Tumor endothelium undergoing endothelial-mesenchymal transition is one of the major sources of CAFs via transdifferentiation. One of the pro-metastatic functions of CAFs involves the promotion of angiogenesis, as demonstrated in many solid tumors, including colon cancer [30], melanoma, and pancreatic tumors [31,32], triple-negative breast cancer [33], hepatocellular carcinoma [34], gastric cancer [35], and oral squamous cell carcinoma [36] culminating into tumor progression [37]. Secondly, CAF plays a critical role in the development of anti-angiogenic therapy, especially anti-VEGF treatment in solid tumors [22,38]. Indeed CAFs have been proposed as “Trojan Horse” mediators of resistance to anti-VEGF therapy [39]. In ovarian cancers, several hypotheses regarding the CAF’s origin and its functions in modulating the structure and composition of the TME in the course of the evolution of tumors have been proposed, with a view of potentially targeting CAFs as a possible therapeutic target [40,41]. However, a direct cause-effect relationship between the CAF function and angiogenesis has not been experimentally proven.

Considering the role of CAFs in tumor angiogenesis and the development of resistance to anti-angiogenic drugs in ovarian neoplasms, we hypothesized that ovarian CAF plays a critical role in resisting anti-angiogenic effects via direct crosstalk with endothelium. Our HyCC model of Patient-Derived ex vivo primary CAFs and HUVEC endothelial cells tested our hypothesis to prove that CAFs impart resistance to anti-angiogenic drugs. For the first time, we proved the ability of Patient-Derived primary CAFs to resist lenvatinib’s effect. Our HyCC model can be used to evaluate the crosstalk of Patient-Derived CAFs with endothelium during the course of developing resistance to anti-angiogenic drugs in ovarian neoplasms well before it is encountered in clinics. 

## 2. Methods and Materials

### 2.1. Patients and Tissue Collection at the Time of Surgery

All experimental protocols were approved by the institutional and/or licensing committee(s). The informed consent(s) (IRB approved: Protocol Number Study: 2017.053-100399_ExVivo001) was obtained from 27 subjects and/or their legal guardian(s). Tissue samples were collected in designated collection media during the time of surgery in accordance with the guidelines of the pathology department in accordance with relevant guidelines and regulations. All methods were carried out in accordance with relevant guidelines and regulations. We included samples from consecutive consented patients with ovarian tumors at any stage/grade of the disease undergoing surgery with or without pre-treatment/history of any previous carcinoma. Both tumor and tumor-adjacent normal tissue were obtained as provided by the pathologist, depending upon the availability of the tissue on a case-to-case basis.

### 2.2. Cell Lines and Reagents

Human uterine fibroblasts (HUF; Primary Uterine Fibroblasts, Cat # PCS-460-010) and HUVEC cells were procured from ATCC (cat # PCS-100-013) and were cultured according to the standard cell culture procedures as per ATCC. Other cell lines for qRT-PCR were procured from ATCC. Matrigel was procured from BD biosciences, USA. Fluorescence vital stains, DiI, and DiO were bought from Molecular Probes, USA. Lenvatinib was procured from Selleckchem, PA, USA. Fibroblasts Antibody (TE-7) and recombinant Anti-S100A4 antibodies were procured from NOVUS and Abcam, respectively. PD-L1 [Clone 22C3] was bought from Agilent-Dako. PD-L2 (D7U8C) and CD31 were procured from Cell Signaling, USA. Cytokeratin 8 and 18 (B22.1 and B23.1), Ep-CAM/Epithelial Specific Antigen (Ber-EP4), Actin, Smooth Muscle (1A4), and Vimentin (SP20) were bought from Cell Marque, USA. Primers for qRT-PCR (Primer pairs are ordered from Integrated DNA Technologies, Inc. [IDT], Iowa, USA) of primary CAF are presented below:

### 2.3. Gene Primer Sequences Used for the Study (Sequences Listed 5′-3′) Are Listed Below

ACTA-2/SMA
  F: CGT TAC TAC TGC TGA GCG TGA
    R: GCC CAT CAG GCA ACT CGT AA

CD31
  F: ATT GCA GTG GTT ATC ATC GGA GTG
    R: CTG GTT GTT GGA GTT CAG AAG TGG

CD44
  F: AGC ACT TCA GGA GGT TAC ATC T
    R: CTT GCC TCT TGG TTG CTG TCT

CD45
  F: CTT CAG TGG TCC ATT TGG TG
    R: CCC TTT GTT CTC GGC TTC CAG

CD90/THY1
  F: GAA GGT CCT CTA CTT ATC CGC C
    R: TGA TGC CCT CAC ACT TGA CCA G

EpCAM
  F: AGC GAG TGA GAA CCT ACT GGA
    R: CGC GTT GTG ATC TCC TTC TGA

FAP-A
  F: GGA AGT GCC TGT TCC AGC AAT G
    R: TGT CTG CCA GTC TTC CCT GAA G

GAPDH
  F: TCA AGG CTG AGA ACG GGA AG
    R: CGC CCC ACT TGA TTT TGG AG

PDGFRA
  F: TGG CAG TAC CCC ATG TCT GAA
    R: CCA AGA CCG TCA CAA AAA GGC

PD-L1
  F: ACC TAC TGG CAT TTG CTG AAC G
    R: ATA GAC AAT TAG TGC AGC CAG GT

S100A4
  F: CAG AAC TAA AGG AGC TGC TGA CC
    R: CTT GGA AGT CCA CCT CGT TGT C

### 2.4. Expression of Tumor Cell Proliferation, Apoptotic Markers, and CAF Markers in the Tumor Sample

Tumor samples were collected at the time of surgery/biopsy as a part of a protocol approved by the institutional and/or licensing committee. We included tumor samples from patients with ovarian neoplasms at any stage/grade of the disease undergoing surgery/biopsy with or without pre-treatment/history of any previous carcinoma. We did not include any bone-marrow transplant patients or patients with liquid tumors. We tested the expression of Ki67, cl-caspase3 (cl-c3), and cl-PARP in representative tumor samples from patients with ovarian cancers by dual IHC stain for Ki67 (visualized by brown DAB color) and cl-c3 (visualized by pink alkaline phosphatase color) as well as cl-PARP (visualized by brown DAB color). The morphological features are presented by H&E stain. The expression of markers for angiogenic blood vessels (CD31) and fibroblast/CAFs (alpha SMA, TE-7, S100A4) was tested by IHC along with the expression of proliferation (Ki67), apoptotic markers (cl-PARP and cl-c3) in the tumor sample at day zero. The expression of PD-L1, PD-L2, and PD-1 was also tested both in the tumor and TME components. 

### 2.5. Cord Formation Assay in HUVEC Cells

The cord formation assay was performed, as reported earlier [42]. In short, HUVEC (Human Umbilical Vein Endothelial Cells) cells were plated on growth factor reduced phenol red free matrigel in 6–8 replicates. Pictures of cells were taken at zero and four, and 18 h. HUVEC cords were stained with hematoxylin and PAS, as mentioned before [42]. As we reported earlier, the cord formation was confirmed by testing the effect of bevacizumab which abrogated the cord formation of HUVEC cells [22]. The phenotypic performance of HUVEC cells in forming cords on matrigel was tested before and after staining by measuring the angiogenic index. 

### 2.6. Quantification of Angiogenic Event

The angiogenic index was calculated based on the angiogenic scores as described elsewhere [10] with modifications. Two parameters indicative of the vasculogenic capacity of HUVEC cells, meshes, or complete polygons and polygonic junctions, were assessed manually in a semi-quantitative manner. The complete polygons are defined as the formation of polygonal structures (meshes) made by HUVEC cells following 4–6 h of plating on growth factor reduced phenol red free matrigel. The % of formation of polygonal structures in 10 random microscopic fields at a particular magnification was expressed. Junctions are defined as multicellular aggregates of cells at the meeting point of sides of a completed polygon. The angiogenic “Endpoints” (total number)(represented in Divot bars in Figures) and the “Mean E Lacunarity” (represented in 40% filled bars) were detected with the Angio-Tool software 0.5 (https://ccrod.cancer.gov/confluence/display/ROB2/Home) (access on 9 August 2022) [43]. Being an automated assessment, AngioTool reduces the subjectivity and the probability of human error as well as streamlines the analysis of features compared to the manual evaluation, such as counting the number of complete polygons, endpoints, or junctions per image. Student’s *t*-tests determined statistical significance.

### 2.7. Primary Culture of Patient-Derived Ovarian CAFs

Primary cultures of CAFs derived from the ovarian tumor samples obtained from participating patients were set up from the feeder layer, which was set up from the tumor and tumor-adjacent normal. The initial seeding of cells was cultured in 10%FBS (0.1 μM Sterile Filtered) DMEM/F-12 supplemented with Glutamax, 1% HyClone Penicillin-Streptomycin, Bovine Serum Albumin, and HEPES buffer. Every day, culture reports were collected and filed to provide date-wise details of the passage and to document the passage-wise growth pattern of CAF. Depending on the availability of the CAFs in primary culture, we performed characterization of CAFs in each passage in each patient. The purity and the extent of epithelial cell contamination of the cultures were monitored by testing the expression of mRNA by qRT-PCR as well as protein expression by flow cytometry, Western blots, and Immunocytochemistry (ICC). A passage of the primary culture of CAF is qualified by (1) the negative expression of non-CAF markers, including CK8,18 19, EpCAM epithelial cell markers, leucocyte common antigen CD45, and endothelial cells marker, CD31, and (2) the positive expression of fibroblast/CAF markers, including SMAalpha, S100A4, CD90, FAP, TE-7, and PGDFRa. Expression of stem cell marker, CD44, and immune checkpoint marker, PD-L1, were also monitored. The expression of fibroblast markers was monitored throughout each passage, from early to late, depending on individual patient samples. The first 3 passages are defined as early, followed by mid and late passages. Depending upon the viability and expression of markers, the late passage CAFs have been tested for senescence by beta-galactosidase assays.

### 2.8. Expression of mRNA for CAF Markers by qRT-PCR

RNA extraction was performed using the Qiagen RNEasy MiniKit and Qiashredder Kit according to the manufacturer’s (Qiagen, Germantown, MD, USA) protocol. Briefly, cells were released from culture and pelleted by centrifugation at 1500 rpm for 5 min. Cells were placed in a lysis buffer and lysed using the Qiashredder system. RNA was then extracted using appropriate reagents. RNA was converted to cDNA using iScript Reverse Transcription Supermix. qRT-PCR was performed using the Roche LightCycler96 platform. In brief, appropriate primers for each gene of interest were mixed with Roche FastStart Essential DNA Green Master Mix and added to a 96-well plate. Each sample was run in triplicates (Roche LightCycler 96 Software version 1.1). Appropriate primers for each gene were ordered from Integrated DNA Technologies.

### 2.9. Expression of CAF Marker Proteins by Flow Cytometry and Western Blot

Flow cytometric analyses were performed as mentioned elsewhere [44]. In brief, cells were trypsin released and rinsed in FACS buffer (1% FBS in phenol red-free RPMI). Cell number was adjusted to 10^6^ cells per sample, resuspended in FACS buffer along with corresponding cell surface antibodies (FAP-PE R&D systems, FAB3715P; and FAP-AF488 R&D systems FAB3715G, PD-L1: 130-122-810 from Miltenyi), and incubated at 4 °C for 20 min (FAP-FITC; SMA-PE, Novus NBP2-34760PE; S100A4-PerCP: NBP2-54580PCP; PD-L1-APC: 130-122-810). Cells were rinsed twice with FACS buffer, then fixed for 30 min, rinsed and resuspended in permeabilization buffer (Fix/Perm kit Miltenyi), and the corresponding intracellular antibodies were added for 30 min (SMA: NBP2-34760F and S100A4: NBP2-54580PCP, Novus). Cells were rinsed, resuspended in FACS buffer, and analyzed on the Accuri C6 flow cytometer. FCS Express was used for analysis. The expression of CAF marker proteins, including SMA (Mouse monoclonal, CellMarque Antibodies, 202M94), S100A4 (Rabbit monoclonal, abcam, ab124805), TGFB (Rabbit monoclonal, Cell Signaling, S3709), CD44 (Mouse monoclonal, Cell Signaling, S3570), and PD-L1(Rabbit monoclonal, Cell Signaling, S13684), were tested by conventional Western blot analyses as described elsewhere [45]. Beta-actin (Mouse monoclonal, Sigma-Aldrich, A2228) was used as the loading control for Western blots. 

### 2.10. Cellular Localization of CAF Markers by ICC

Primary ovarian CAFs were stained for both positive (SMA, TE-7, and S100A) and negative (CK 8,18 and CD31) markers following formaldehyde fixation. We used standard DAB-based ICC for identifying the cellular localization of the marker proteins. The frozen CAFs were thawed and tested for the same markers. CAFs were stained with H&E for morphological reference. 

### 2.11. Ex Vivo Hybrid Co-Culture of Patient-Derived Primary Ovarian CAF and HUVEC Cells

The HyCC of Patient-Derived primary CAF and HUVEC cells was set up using qualified CAFs from the early passages of the primary culture. HUVEC cells were stained with fluorescent red-DiI stain for the co-culture, and primary CAFs were stained with the fluorescent green-DiO stain according to the manufacturer’s method. A standard flow cytometric assay tested the efficiency of staining of both cells. The viability of 2 types of cells was separately tested after staining. The HyCC is a cell-to-cell contact co-culture and was carried out in sequential and simultaneous formats. For the sequential HyCC, DiO-stained qualified primary CAFs (passage 1) from ovarian tumor tissue were plated on the growth factor reduced phenol red free matrigel in 6-8 replicates 24–48 h before the addition of DiI-stained HUVEC cells (passage 4) on the top of plated CAFs. HUVEC cells were added with or without lenvatinib. For the simultaneous HyCC, DiO-stained qualified primary CAFs (passage 1) from ovarian tumor tissue were plated on the matrigel along with DiI-stained HUVEC cells (passage 4) on the top of growth factor-reduced phenol-red free matrigel in 6–8 replicates with or without lenvatinib. Pictures of cells were taken at zero hours, 4, and 18 h. For both the sequential and simultaneous HyCC, a parallel set of experiments was set up in a well without matrigel as an internal negative control.

### 2.12. Statistical Analysis

All in vitro experiments were performed at least three times independently in triplicates. Student’s *t*-test is used to evaluate differences observed between treated groups and vehicle-treated controls. The data are presented as means ± SE and were analyzed by unpaired two-tailed Student’s *t*-test. A *p*-value of *p* < 0.05 was considered statistically significant.

## 3. Results 

### 3.1. Expression of Different Markers in the Representative Tumor Samples of Ovarian Cancers

**Figure 1:**
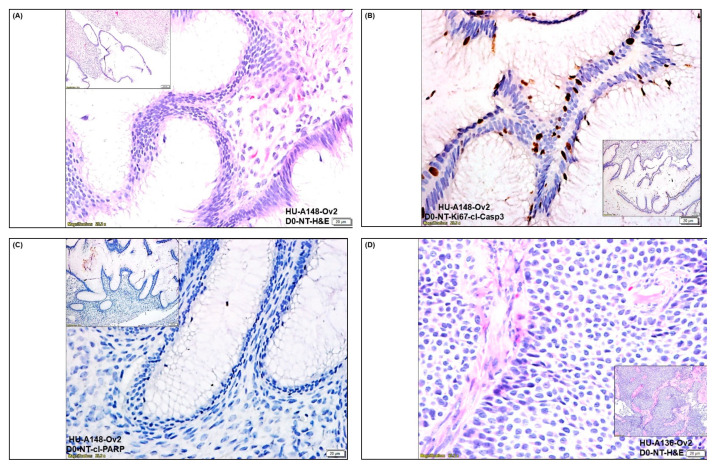

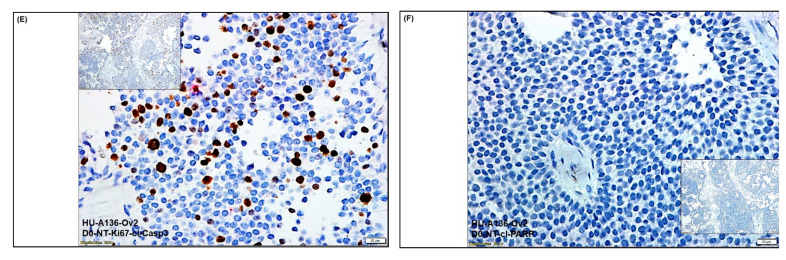
**Expression of tumor cell proliferation and apoptotic markers in the representative tumor samples of ovarian cancers:** We tested the expression of Ki67, cl-Casp3, and cl-PARP in two representative tumor samples from patients with ovarian cancers HU-A148-Ov2 and HU-A136-Ov2 with grade 1, stage IVA low-grade appendiceal mucinous neoplasm (pT4b pN0 pM1b) (**B**,**C**), and with stage IA adult granulosa cell tumor (pT1a NX) (**E**,**F**) by dual IHC stain for Ki67 (visualized by brown DAB color), and cl-Casp3 (visualized by pink alkaline phosphatase color) (**B**,**E**) as well as cl-PARP (visualized by brown DAB color) (**C**,**F**). The morphological features are presented by H&E stain (**A**,**D**).

**Figure 2:**
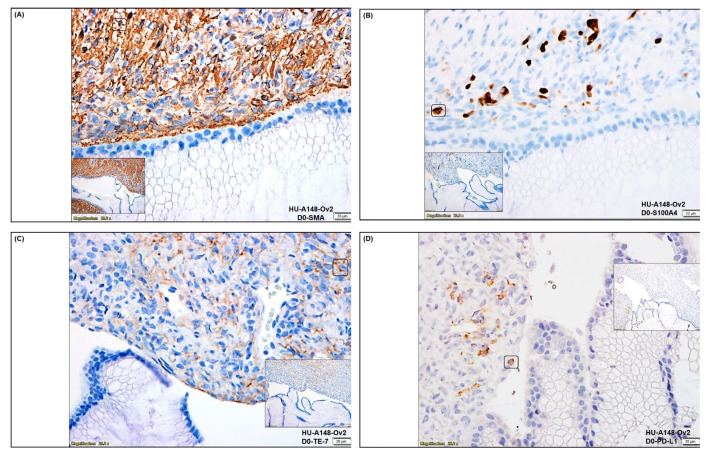

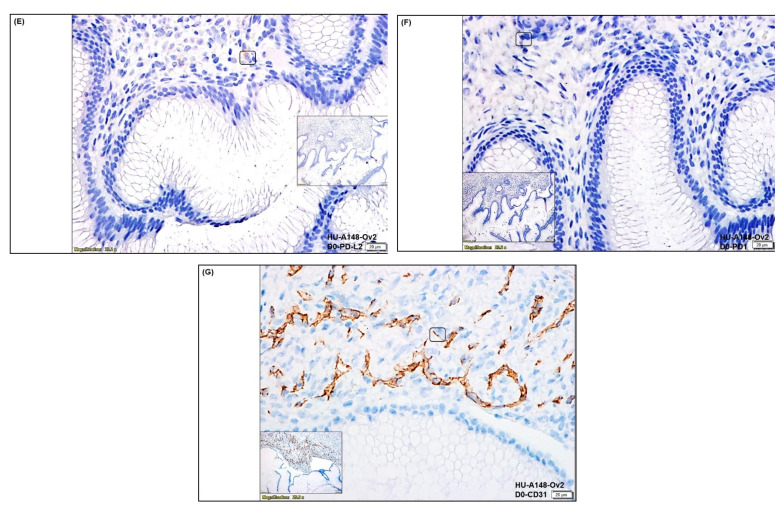
**Expression of positive and negative CAF markers, Immune markers, and angiogenic markers in the representative tumor sample of ovarian cancers:** We tested the expression of SMA (**A**), S100A4 (**B**), TE-7 (**C**), PD-L1 (**D**), PD-L2 (**E**), PD-1 (**F**), and CD31 (**G**) in tumor samples from patients with ovarian cancers by IHC.

We tested the expression of tumor cell proliferation markers (Ki67) and apoptotic markers (cl-c3 and cl-PARP), along with positive CAF markers (SMA, S100A4, TE-7), negative CAF markers (CK, 8,18, EpCAM, CD45), immune markers (PD-L1, PD-L2, and PD-1), and angiogenic marker (CD31) in the representative tumor sample of ovarian cancers. Figure 1 presents the expression of Ki67 and cleaved caspase3 (cl-c3) on the same FFPE sections (Figure 1B,E) and cleaved PARP (cl-PARP) (Figure 1C,F) from the tumor samples of 2 patients one with grade 1, stage IVA low-grade appendiceal mucinous neoplasm (pT4b pN0 pM1b), and other with stage IA adult granulosa cell tumor (pT1a NX). The dual stain for Ki67 and cl-c3 of the tumor sample from the patient with grade 1, stage IVA low-grade appendiceal mucinous neoplasm demonstrated moderate staining for the Ki67 with no positive stain for cl-c3 (Figure 1B). Dual staining for Ki67 and cl-c3 of the tumor sample from the patient with stage IA adult granulosa cell tumor showed a few cl-c3 positive cells with frequent Ki67 positive tumor cells (Figure 1E). The staining for cl-PARP was negative in both tumor samples (Figure 1C,F). Since CAFs were established from the tumor tissue, we tested the expression of SMA, S100A4, TE-7, PD-L1, PD-L2, and PD-1 in the tumor tissue obtained from the patient with grade 1, stage IVA low-grade appendiceal mucinous neoplasm (Figure 2A–F). The tumor tissue was found highly positive for the expression of positive CAF markers, including SMA, S100A4, and TE-7 (Figure 2A–C). The tissue was also found positive for PD-L1 expression with a very rare positive stain for PD-L2 and negative for PD-1 stain (Figure 2D–F). To test the vascularity of the tumor tissue in the context of tumor-induced vascularization’s role in tumor growth, we also stained the tumor tissue sample with CD31 and observed a moderately high degree of stain (Figure 2G).

### 3.2. Expression of CAF Markers in Established Ovarian CAF

**Figure 3:**
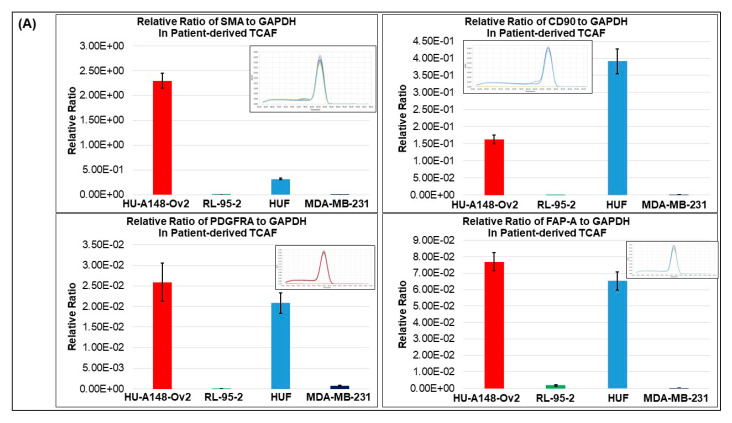

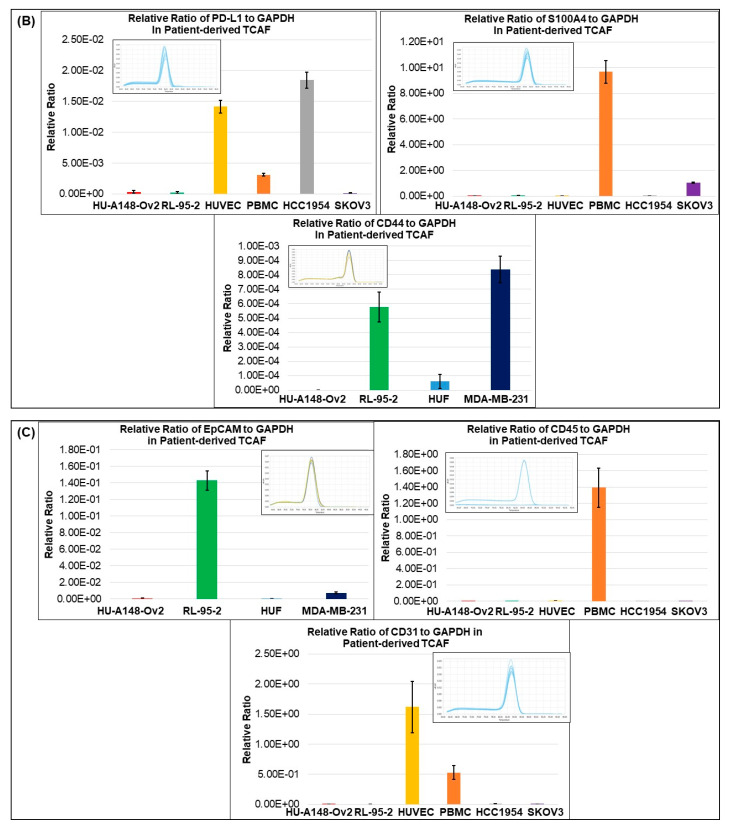

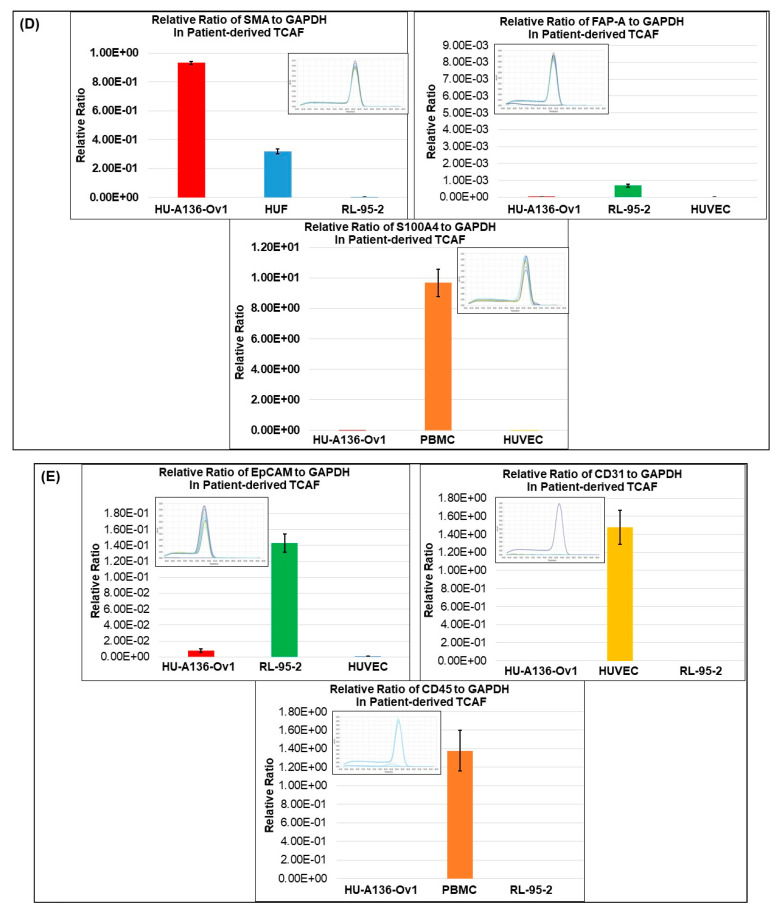

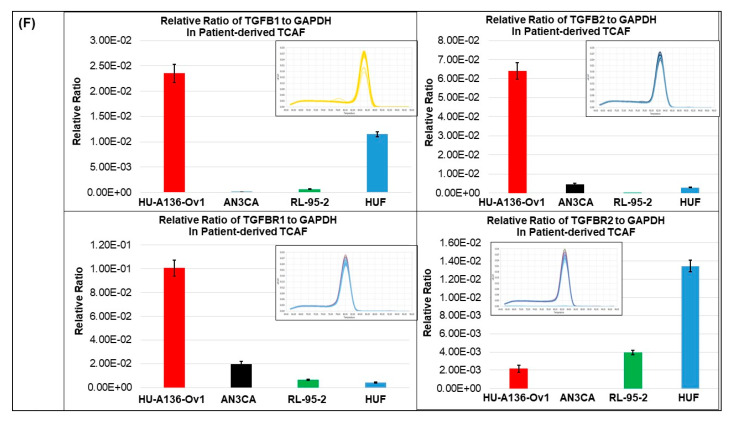
**Expression of mRNA for positive and negative CAF markers in the Patient-Derived primary culture of ovarian CAF established from the representative tumor sample of ovarian cancers:** We tested the expression of mRNA for SMA, CD90, PDGFRA and FAP-A (**A**), PD-L1, S100A4, and CD44 (**B**), EpCAM, CD45, and CD31 (**C**) in the tumor-derived CAF from the patient with grade 1, stage IVA low-grade appendiceal mucinous neoplasm and SMA, FAP-A, and S100A4 (**D**), EpCAM, CD45, and CD31 (**E**) and TGFB1, TGFB2, TGFBR1 and TGFBR2 (**F**) in the tumor-derived CAF from the patient with stage IA adult granulosa cell tumor. Bars in red color represent CAF derived from the tumor tissue, bars in green color represent the RL-95-2 endometrial cancer cell line, bars in blue color represent HUF, bars in dark blue color represent the MDA-MB-231 breast cancer cell line, bars in mustard color represent HUVEC endothelial cell line, bars in ash color represents HCC1954 breast cancer cell line, bars in orange color represents human PBMC, bars in dark violet color represents SKOV3 ovarian cancer cell line, bars in black color represents AN3CA endometrial cancer cell line. The melting curves are presented as the inset in each bar diagram.

**Figure 4:**
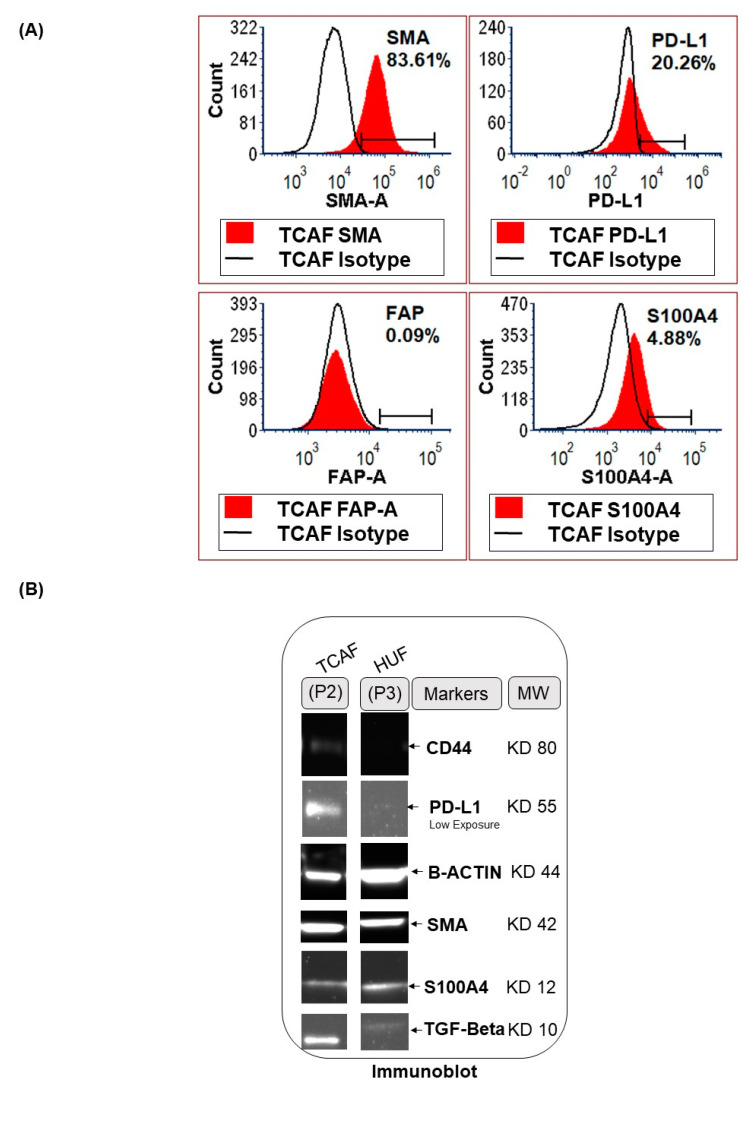
**Expression of proteins for CAF markers in the Patient-Derived primary culture of ovarian CAF established from the representative tumor sample of ovarian cancers:** We tested the expression of protein by flow cytometry for SMA, PD-L1, S100A4, and FAP-A (**A**), and the expression of protein by Western blot for SMA, PD-L1, S100A4, TGFB, and CD44 (**B**) in the Patient-Derived CAFs. B-actin was used as the loading control for the Western blot.

**Figure 5:**
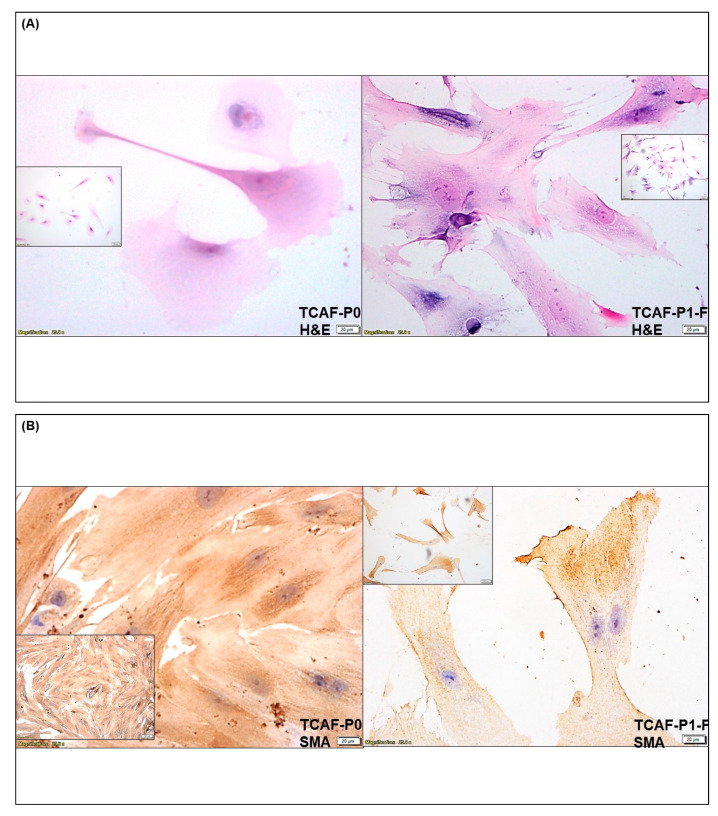

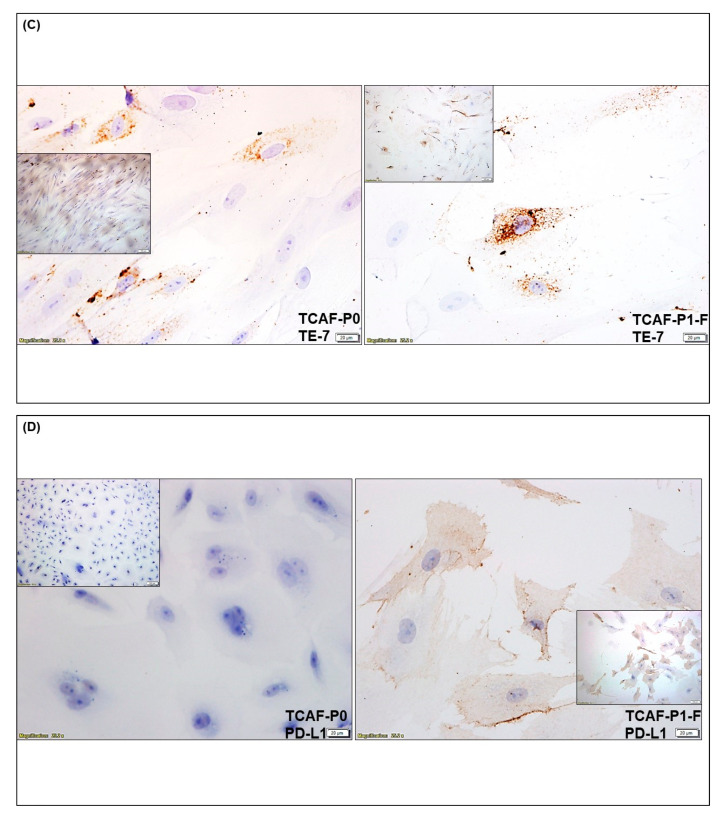

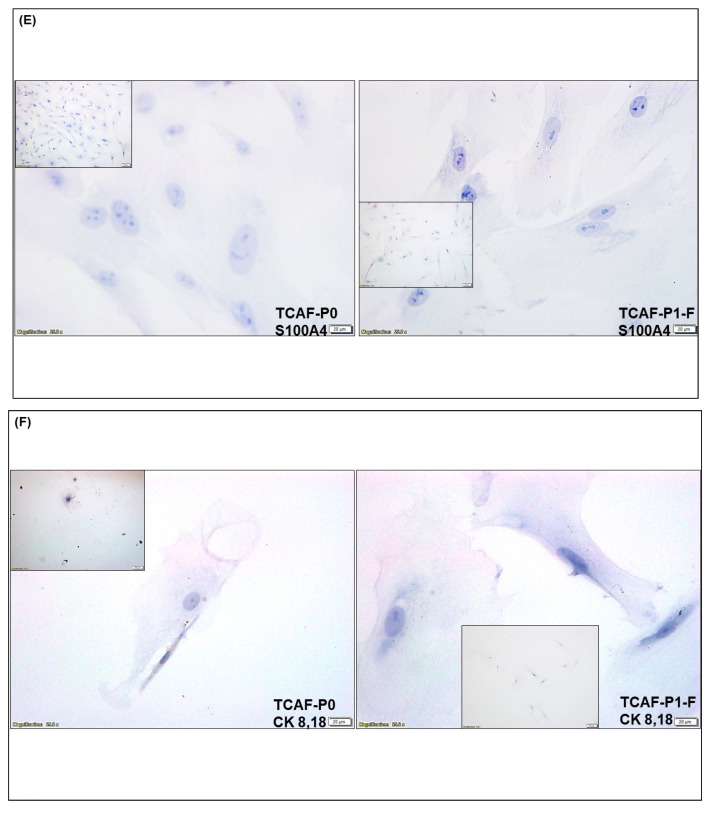

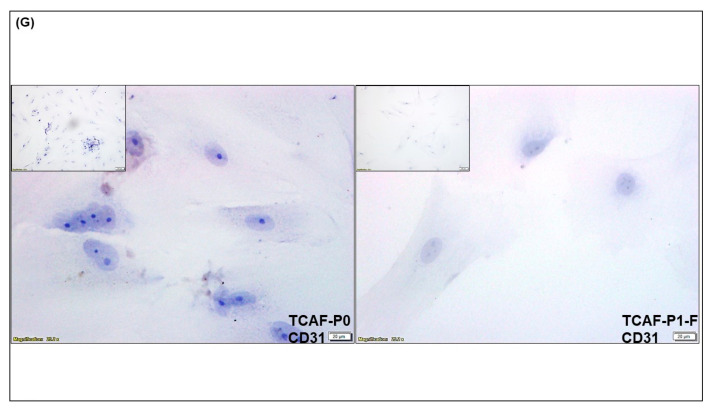
**Cellular localization of proteins for CAF markers in the Patient-Derived primary culture of ovarian CAF from pre- and post-frozen passages established from the representative tumor sample, HU-A148-Ov2 of ovarian cancers:** We tested the cellular localization of the expression of protein by ICC for SMA (**B**), TE-7 (**C**), PD-L1 (**D**), S100A4 (**E**), CK 8, 18 (**F**), and CD31 (**G**) in Patient-Derived CAFs (before and after thawing). The morphological features are presented by H&E stain (**A**). Insets represent photomicrographs in lower magnification.

CAFs were established from both tumor and the tumor-adjacent normal tissues received at the time of surgery, depending on the availability of tissue samples. The characterization was performed by the expression of several positive, negative and functional markers of CAF. Ovarian cancer CAFs were characterized by negative expression of cell type markers, including leucocyte common antigen CD45, macrophage marker CD68, endothelial marker CD31, and epithelial marker EpCAM with parallel positive expression of fibroblast markers, SMA, FAP, S100A4, and TE-7 (flow cytometry, qRT-PCR, Western blot, and ICC). PD-L1 and CD44 were used separately. Figure 3 presents the expression of mRNA for the protein markers of CAF in the Patient-Derived primary CAFs from two representative ovarian tissues in our study cohort. CAFs from both patients were positive for SMA (Figure 3A,D), while both CAFs were negative for S100A4 (Figure 3B,D). The CAF derived from the resected tumor sample of the patient with grade 1 and stage IVA low-grade appendiceal mucinous neoplasm demonstrated high levels of FAP-A, while negligible levels of FAP-A mRNA were found in the CAF of the patient with stage IA adult granulosa cell tumor. CAFs from both patients’ tumors expressed no S100A4 mRNA (Figure 3B,D). We detected mRNA for CD90 and PDGFRA, but a negligible amount of PD-L1 in CAFs derived from the patient with grade 1, stage IVA low-grade appendiceal mucinous neoplasm (Figure 3A,B). None of the CAFs expressed CD45, CD31, and EpCAM (Figure 3C,E). CAFs derived from the patient with stage IA adult granulosa cell tumor were positive for TGFB1, TGFB2, TGFBR1, and TGFBR2 mRNA (Figure 3F). We tested protein expression by flow cytometry in the Patient-Derived CAF for a few of the proteins whose mRNA expressions have been tested by qRT-PCR. We observed a comparable high expression pattern (for mRNA and protein) of SMA and negligible expression of S100A4 (Figure 3 and Figure 4A). Figure 4B demonstrates the expression of several CAF markers by Western blot analysis. A higher expression of SMA was observed in the CAF sample in contrast to low amounts of S100A4, PD-L1, and TGFbeta. Importantly, CAFs did not express CD44 (Figure 4B). Similarly, we tested the subcellular localization of the expressed protein in CAF by ICC before and after the first freeze–thaw cycle of the CAF (designated as “TCAF-P1-F” in Figure 5) as compared to CAFs from the passage zero (designated as "TCAF-P0" in Figure 5). In addition to the expression of TE-7 (Figure 5C), CAFs were positive for SMA (Figure 5B) and negative for S100A4 (Figure 5E), CK 8,18 (Figure 5F), and CD31 (Figure 5G).

In summary, we tested the expression pattern(s) of several positive (SMA, S100A4, TE-7, CD90, FAP-A, and PDGFRA) and negative (EpCAM, CK8, 18, CD45, and CD31) CAF markers and markers associated with CAF functions (TGFB1, TGFB2, TGFR1, TGFR2, and CD44), as well as immune markers (PD-L1, PD-L2, and PD-1) in the primary CAFs as cultured from resected tumor samples of two patients using four methods, qRT-PCR (Figure 3), flow cytometry (Figure 4A), Western blot (Figure 4B), and ICC (Figure 5). The purpose of the staining was to compare the expression of some these markers, (1) in the founder tumor tissue sample from the resected tumor with their expression in the tumor-derived CAFs from the same patient, and (2) in testing the mRNA expression (by qRT-PCR), protein expression (by flow cytometry), and subcellular expression (by ICC) in different patients. When compared with the expression of some of these markers in the founder tumor tissue sample from the resected tumor (Figure 2), we demonstrated that in an individual patient, the expression pattern of positive CAF markers, including SMA, S100A4, TE-7, and immune markers including PD-L1, PD-L2, and PD-1 in the mesenchyme of the tumor microenvironment was quantitatively similar to the expression in the primary CAFs derived from the tumor tissue. For example, a high (100% positive) expression of SMA was observed in both the tumor mesenchyme by IHC and the tumor-derived CAFs by ICC (compare Figure 2A and Figure 5B left and right panel). Similarly, a high (60% positive) expression of TE-7 was observed in both the tumor mesenchyme by IHC and the tumor-derived CAFs by ICC (compare Figure 2C and Figure 5C left and right panel). In contrast, a low expression of S100A4 was observed in the tumor mesenchyme by IHC (10% positive), which was comparable to the ICC expression (<1% positive) of the same markers in the tumor-derived CAFs (compare Figure 2B and Figure 5E left and right panel). A low (5–10% positive) expression of immune marker PD-L1 was observed in both the tumor mesenchyme by IHC and the tumor-derived CAFs by ICC (compare Figure 2D and Figure 5D). In tumor-derived CAFs from both the patients, we observed high expression of SMA mRNA and protein (100% by ICC and 84% by flow) while a low expression of S100A4 mRNA and protein (1% by ICC and 5% by flow) (Figure 3, Figure 4 and Figure 5). The low mRNA expression of PD-L1 of CAF in one patient was matched to 5% expression by ICC. In contrast, the expression of CD31 and PD-1 was not detected by any methods of detection in both tumor tissue and CAFs. 

### 3.3. Angiogenic Function of Patient-Derived Primary CAFs in Ovarian Cancers Using an Ex Vivo Hybrid Co-Culture Model

**Figure 6:**
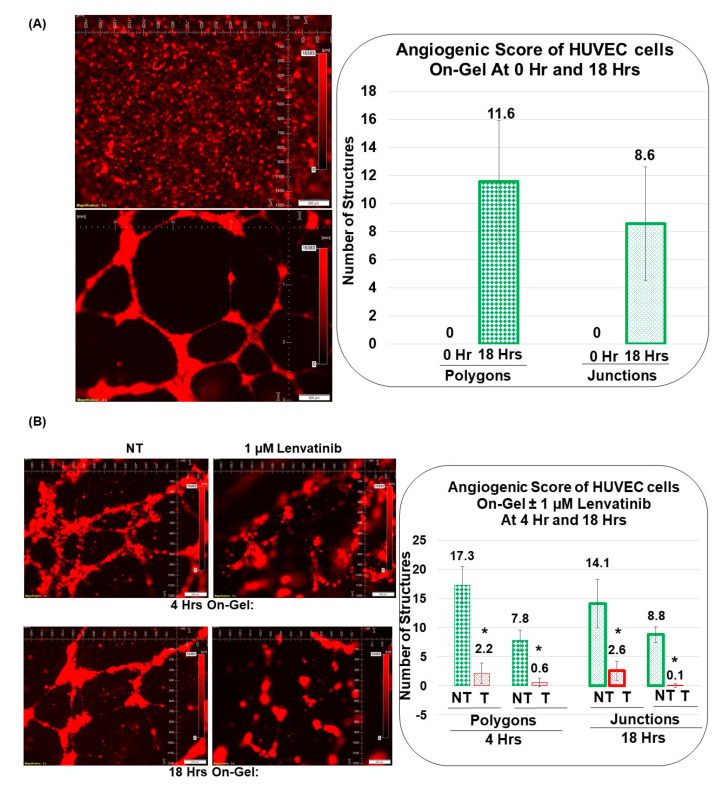

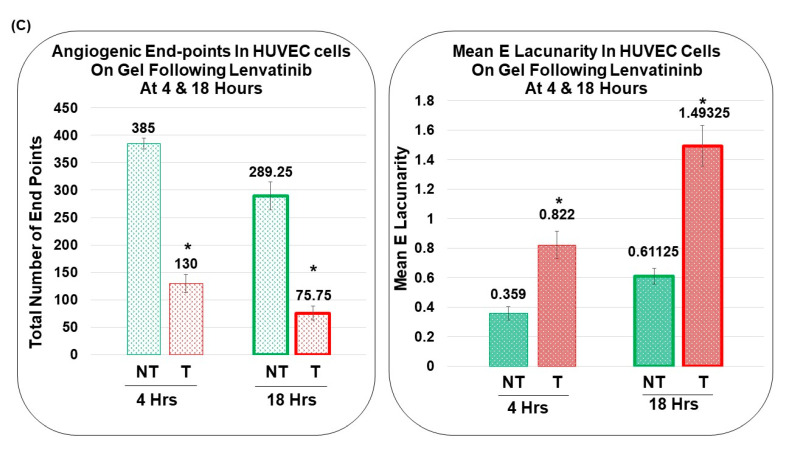
**Effect of Lenvatinib on cord formation of HUVEC cells on−gel:** The effect of lenvatinib on the HUVEC cell cord formation on matrigel is tested at 0 h, 4 h, and 18 h. The angiogenic score of HUVEC cell on-gel cord formation at 0 h and 18 h (**A**) is measured by the number of polygons and junctions. Similarly, the effect of lenvatinib on the angiogenic score of HUVEC cell on-gel cord formation at 4 h and 18 h are measured by the number of polygons and junctions (**B**) as well as measuring angiogenic endpoints and mean E lacunarity (**C**). Matrigel tube formation assay of HUVECs cultured in control medium (green) or in the presence of 1 microM of lenvatinib (red) for 4 h (standard border of the width of bars) and 18 h (thicker border of the width of bars). Angiogenic Endpoints (total number) (represented in Divot bars) and the mean E lacunarity (represented in 40% filled bars) were detected with the *AngioTool* software (https://ccrod.cancer.gov/confluence/display/ROB2/Home; [accessed on 9 August 2022]). Bars represent the mean, and standard deviation of the mean; * *p* values are indicated for significant changes. Please note that the angiogenic endpoints in non-treated HUVEC cells showed a decreasing trend at 18 h as compared to 4 h. This is because the cord formation was dissolved and non-detected at later hours, after 24 h. However, the lacunarity of non-treated HUVEC cells showed an increasing trend at 18 h as compared to 4 h because the cells started to coalesce/aggregate as the cord formation started to dissolve. However, the total number of endpoints significantly decreased, and the lacunarity increased significantly following lenvatinib as compared to the respective controls at both 4 and 18 h, which matched with the pattern of decrease of the number of polygons and the junctions of the complete polygons. Being an automated assessment, *AngioTool* reduces subjectivity and the likelihood of human error and streamlines the analysis of features as compared to the manual evaluation, such as counting the number of complete polygons, endpoints, or numbers of junctions per image.

**Figure 7:**
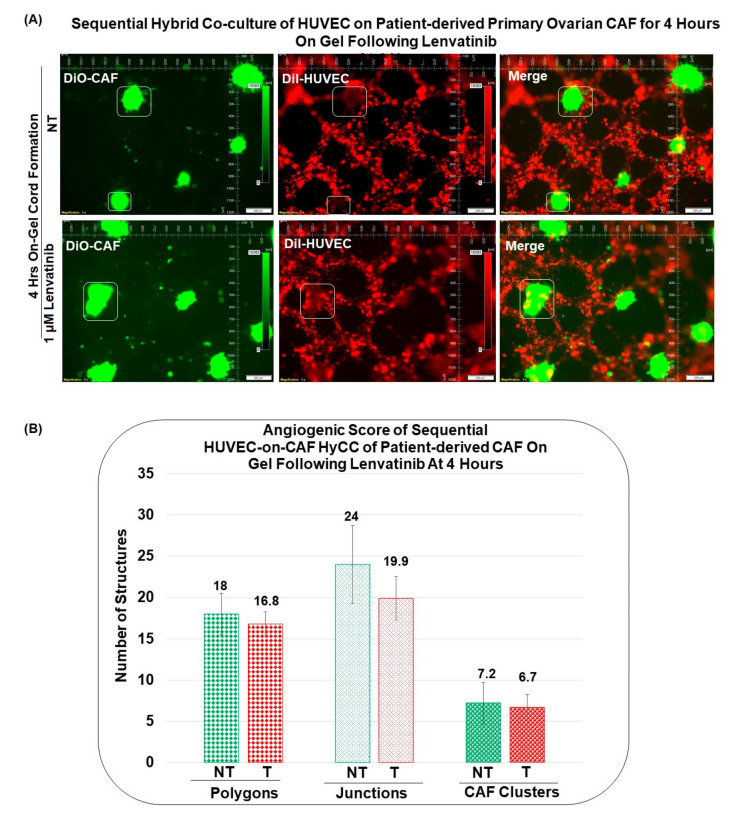

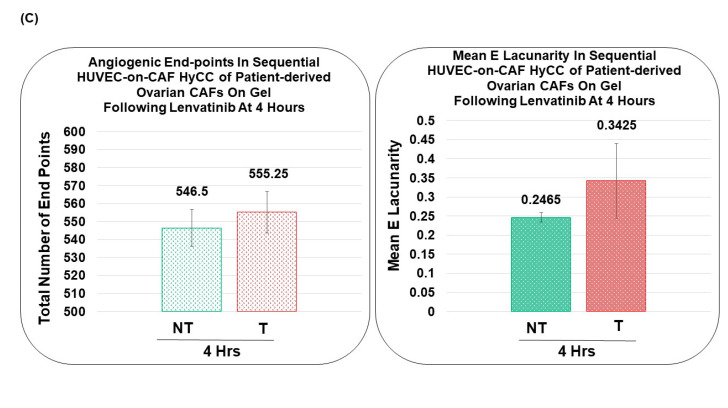
**Effect of Lenvatinib on ex vivo on−gel sequential hybrid co−culture of HUVEC on Patient−Derived primary ovarian CAF for 4 h:** We performed sequential Hybrid Co-Culture of HUVEC on Patient-Derived primary ovarian CAF for 4 h on-gel following lenvatinib (**A**). Angiogenic Scores (**B**) are determined by the polygons, junctions, and CAF clusters (as represented by rectangular/square boxes in photomicrographs of A) as well as angiogenic endpoints and mean E lacunarity (**C**). Matrigel tube formation assay of HUVECs cultured in HyCC format with patient-derived ovarian CAFs in control medium (green) or in the presence of 1 microM of lenvatinib (red) for 4 h. Angiogenic Endpoints (total number) (represented in Divot bars) and the mean E lacunarity (represented in 40% filled bars) were detected with the *AngioTool* software (https://ccrod.cancer.gov/confluence/display/ROB2/Home [(accessed on 9 August 2022)]). Bars represent the mean and standard deviation of the mean.

**Figure 8:**
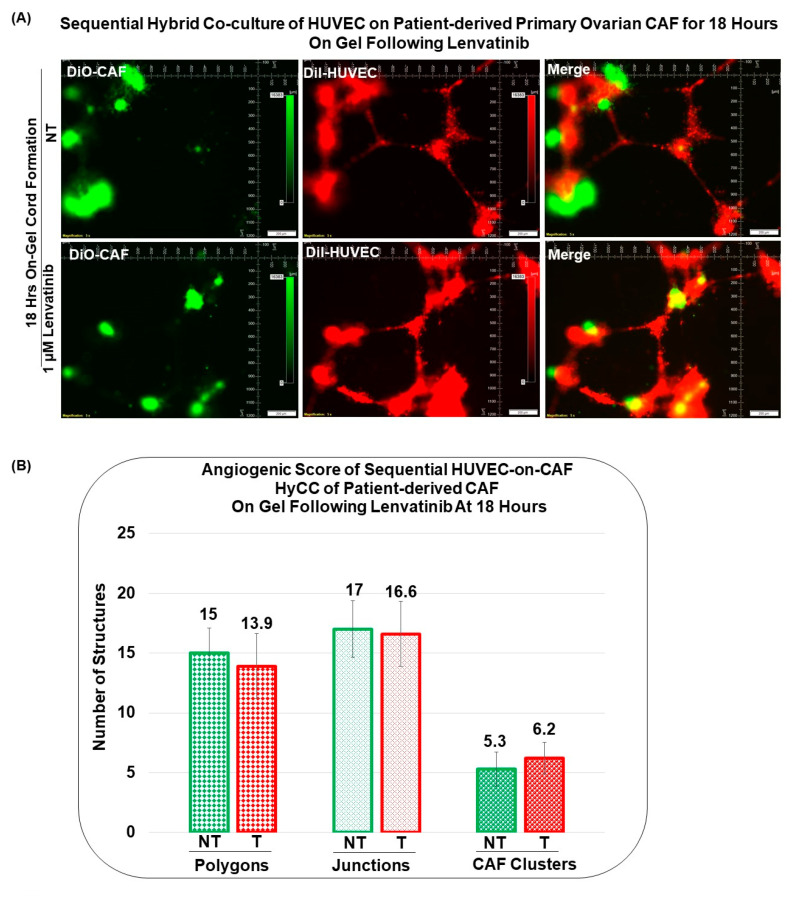

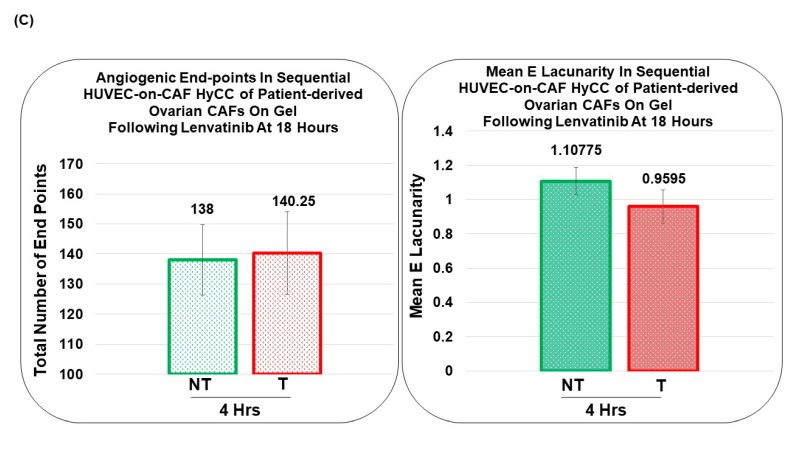
**Effect of Lenvatinib on ex vivo on−gel sequential hybrid co−culture of HUVEC on Patient−Derived primary ovarian CAF for 18 h:** We performed sequential Hybrid Co-Culture of HUVEC on Patient-Derived primary ovarian CAF for 18 h on-gel following lenvatinib (**A**). Angiogenic Scores (**B**) are determined by the polygons, junctions, and CAF clusters as well as angiogenic endpoints and mean E lacunarity (**C**). Matrigel tube formation assay of HUVECs cultured in HyCC format with Patient-Derived ovarian CAFs in control medium (green) or in the presence of 1 microM of lenvatinib (red) for 18 h. Angiogenic Endpoints (total number) (represented in Divot bars) and the mean E Lacunarity (represented in 40% filled bars) were detected with the *AngioTool* software (https://ccrod.cancer.gov/confluence/display/ROB2/Home [(access on 9 August 2022)]). Bars represent the mean and standard deviation of the mean.

**Figure 9:**
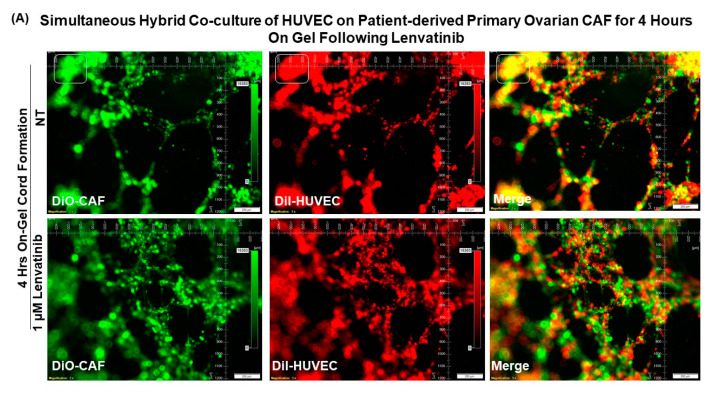

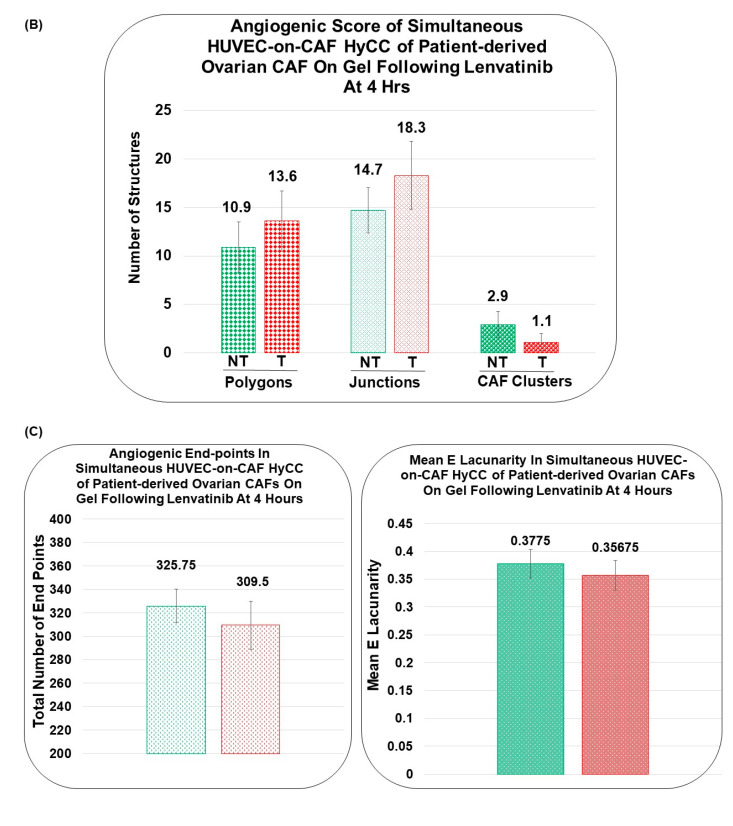
**Effect of Lenvatinib on ex vivo on−gel simultaneous hybrid co−culture of HUVEC on Patient−Derived primary ovarian CAF for 4 h:** We performed simultaneous Hybrid Co-culture of HUVEC on Patient-Derived primary ovarian CAF for 4 h on-gel following lenvatinib. Angiogenic Scores (**B**) are determined by the polygons, junctions, and CAF clusters (as represented by square boxes in photomicrographs of (**A**)) as well as angiogenic endpoints and mean E lacunarity (**C**). Matrigel tube formation assay of HUVECs cultured in HyCC format with Patient-Derived ovarian CAFs in control medium (green) or in the presence of 1 microM of lenvatinib (red) for 4 h. Angiogenic Endpoints (total number) (represented in Divot bars) and the mean E lacunarity (represented in 40% filled bars) were detected with the *AngioTool* software (https://ccrod.cancer.gov/confluence/display/ROB2/Home [(access on 9 August 2022)]). Bars represent the mean and standard deviation of the mean. Please note that the formation of CAF clusters was less in the simultaneous co-culture as compared to sequential co-culture.

A HyCC was established between Patient-Derived primary CAFs and HUVEC cells on matrigel, and cord formation of HUVEC cells on the gel was evaluated at 18 h (Figure 5A). As a reference, cord formation was tested in HUVEC cells following lenvatinib. Cord formations on matrigel by HUVEC cells are inhibited by lenvatinib as quantified using the angiogenic score, angiogenic endpoints, and mean E lacunarity (detected with the Angio-Tool software (https://ccrod.cancer.gov/confluence/display/ROB2/Home; [accessed on 9 August 2022]) at 4 h and 18 h (Figure 6B,C). Lenvatinib had no effect on primary ovarian CAFs either on the plate or on matrigel. Cord formations on matrigel by HUVEC cells occurred on primary ovarian CAFs in two tested formats, sequential HyCC (Figure 7 and Figure 8) and simultaneous HyCC (Figure 9) on matrigel for 4 (Figure 7 and Figure 9) and 18 h (Figure 8). Primary ovarian CAFs protected the cord formations by HUVEC cells on matrigel from the inhibitory effect of lenvatinib (Figure 6B,C). Figure 7 presents that Patient-Derived primary ovarian CAF resists the anti-angiogenic effect of lenvatinib on cord formation of HUVEC cells following Lenvatinib in sequential HyCC CAF on the gel for 4 h. A merge of images (Upper right panel) of DiI-stained HUVEC image of the cord formation (Upper mid panel) without treatment for 4 h on DiO-stained CAF (Upper left panel) showed the differential cord formation of HUVEC cells compared to the co-cultured CAF (Figure 7A). Strikingly, no difference was observed after lenvatinib treatment (Figure 7A Lower panel). The effect was also quantified by the angiogenic score (polygons and junctions), angiogenic endpoints, and mean E lacunarity (detected with the Angio-Tool software (https://ccrod.cancer.gov/confluence/display/ROB2/Home; [(accessed on 9th August 2022)]) (Figure 7B,C). Interestingly we observed that CAFs tend to form a cluster on matrigel (designated as CAF clusters whereof). We observed no change in the number of CAF clusters between non-treated and lenvatinib-treated HyCC. 

Figure 8 presents that Patient-Derived primary ovarian CAF resists the anti-angiogenic effect of lenvatinib on cord formation of HUVEC cells following lenvatinib treatment in sequential HyCC CAF on the gel for 18 h. A merge of images (Upper right panel) of DiI-stained HUVEC image of the cord formation (Upper mid panel) without treatment for 4 h on DiO-stained CAF (Upper left panel) showed the differential cord formation of HUVEC cells compared to the co-cultured CAF (Figure 8A). Similar to the cord formation at 4 h, no difference was observed after lenvatinib treatment (Figure 8A Lower panel) as compared to the non-treated HyCC (Upper panel). The effect was also quantified by the angiogenic score (polygons and junctions), angiogenic endpoints, and mean E lacunarity (detected with the Angio-Tool software (https://ccrod.cancer.gov/confluence/display/ROB2/Home [(access o on 9 August 2022)]) (Figure 8B,C). No change in the number of CAF clusters between non-treated and lenvatinib-treated HyCC was observed. To clarify the effect of CAFs on the on-gel cord formation and resist the effect of anti-angiogenic lenvatinib, we tested the simultaneous HyCC of HUVEC on Patient-Derived primary CAF for 4 h following lenvatinib treatment. Figure 9 presents that Patient-Derived primary ovarian CAF resists the anti-angiogenic effect of lenvatinib on cord formation of HUVEC cells following lenvatinib in simultaneous HyCC CAF on the gel for 4 h. Similar to the sequential HyCC, the HUVEC cells formed a cord on CAFs in a simultaneous HyCC CAF (Figure 9A), irrespective of lenvatinib treatment. The effect was also quantified by the angiogenic score (polygons and junctions), angiogenic endpoints, and mean E lacunarity (detected with the Angio-Tool software (https://ccrod.cancer.gov/confluence/display/ROB2/Home [(accessed on 9tAugust 2022)]) (Figure 9B,C). Interestingly, the formation of CAF clusters was significantly reduced in the simultaneous HyCC. Data from our HUVEC-on-CAF ex vivo HyCC study demonstrate that (1) cord formations on matrigel by HUVEC cells are inhibited by lenvatinib while lenvatinib had no effect on primary ovarian CAFs either on the plate or on matrigel, (2) cord formations on matrigel by HUVEC cells occur on primary ovarian CAFs, and (3) primary ovarian CAFs protected the cord formations by HUVEC cells on matrigel from the inhibitory effect of lenvatinib both in sequential and simultaneous HyCC.

### 3.4. Pro-Angiogenic Function of Patient-Derived Primary CAF in Ovarian Cancers as Tested Using an Ex Vivo Hybrid Co-Culture “On-Plate”

**Figure 10:**
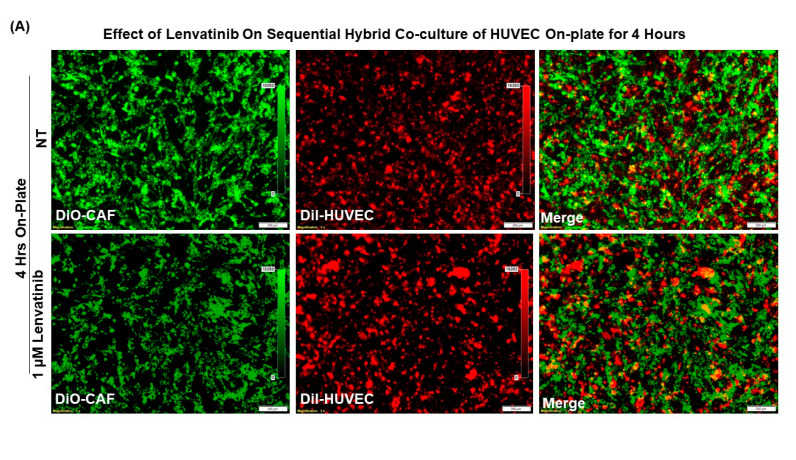

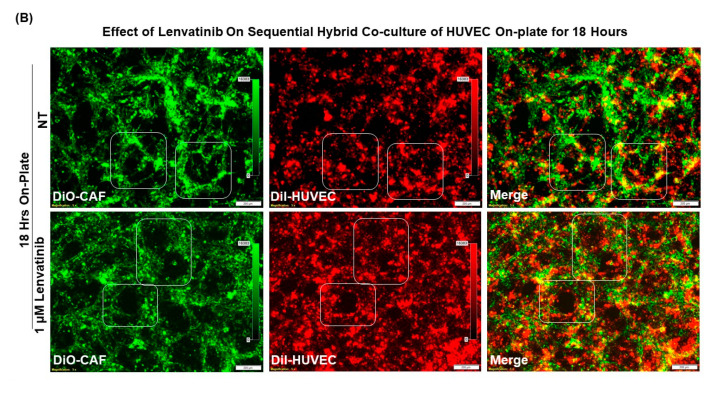
**Effect of Lenvatinib on ex vivo “on−plate” sequential hybrid co−culture of HUVEC on Patient−Derived primary ovarian CAF for 4 and 18 h:** We tested the effect of lenvatinib on sequential Hybrid Co-Culture of HUVEC “on-plate for 4 h (**A**) and for 18 h (**B**). Please note the cord formation at 18 h of co-culture, as shown by square boxes on the photomicrographs.

Since the presence of CAF caused the cord formation of HUVEC cells on gel and protected it from the anti-angiogenic effect of lenvatinib both in sequential and simultaneous HyCC, we tested the effect of CAF in the initiation of cord formation independent of matrigel. Sequential HyCC of HUVEC cells on CAF (cultures “on-plate”) was performed in the presence of lenvatinib (Figure 10). To find if CAF-derived ECM is enough to initiate cord formation in HUVEC without matrigel (“on-plate” only in the sequential HyCC) with “on-plate” only as the technical control, we tested the cord formation at 4 h and 18 h in the presence and absence of lenvatinib. As expected, there was no cord formation at 0 h. No cord formation was observed at 4 h. Interestingly, we noted the cord formation at 18 h of co-culture, as shown by square boxes on the photomicrographs indicating the role of CAF in initiating the cord formation independent of matrigel and protecting the cord formation from the anti-angiogenic effect of lenvatinib. In comparison, no cord formation was ever observed for HUVEC cells “on-plate” without CAF. When plated sequentially on-CAF without matrigel, we observed cord formation of HUVEC cells at 18 h (Figure 10B) as compared to no effect at 4 h (Figure 10A). Interestingly, the rudimentary polygons, junctions, angiogenic-endpoints and lacunarity were observed in the HyCC, and more prominently in the CAFs indicating CAFs direct role in the orchestrating the cord formation. As expected, we observed no cord formation of HUVEC cells “on-plate” at 4 h and 18 h.

## 4. Discussion

In a progressing angiogenic tumor, CAFs are directly involved in the formation of new vessels in various solid tumors. Literature showed that CAFs actively promote tumor angiogenesis by pro-angiogenic signals via secretomic soluble signaling factors, including VEGF, stromal-derived factor1 (SDF-1), YAP, and HIF-1alpha pathways (See [46]). It has been demonstrated that the stromal-derived factor1 (also known as CXCL12), the ligand of CXCR4 secreted from CAFs, recruits endothelial cell precursors to promote angiogenesis [47]. CXCR4 (receptor for stromal-derived factor1), which is highly expressed in the cancer cells, once activated, directly stimulates cancer cell proliferation in breast cancers [48]. Similarly, in invasive breast carcinomas, stromal fibroblasts are shown to promote tumor growth and angiogenesis through elevated SDF-1/CXCL12 secretion [48]. In prostate cancer, the connective tissue growth factor is excessively expressed in CAFs from a differential reactive stroma (DRS) xenograft model that has been shown to promote tumorigenesis and angiogenesis [49]. To promote tumor angiogenesis, CAF exhibits paracrine pro-angiogenic signals and biomechanical properties involving mechanotransductive pathways, which alters their vasculogenic property as tested in vitro 3D microtissue model [46]. Considering the facts that (1) CAFs actively promote tumor angiogenesis and (2) anti-angiogenic drug resistance in ovarian neoplasms is clinically associated with the progression of the disease/poor outcomes, we tested the effect of Patient-Derived primary CAFs in resisting anti-angiogenic drug, lenvatinib-induced abrogation of HUVEC cell cord formation using a HyCC model. This study aimed to investigate the pro-angiogenic function of Patient-Derived ovarian CAF populations via CAFs’ influence on vascular network formation, maturation, and resisting anti-angiogenic drugs on endothelial cells. We chose two of the representative sets of characterized Patient-Derived ovarian CAFs established from our patient cohort to demonstrate that the presence of CAFs conferred protection from lenvatinib-induced inhibition of endothelial cell cord formation in HyCC. We report experimental evidence for a pro-angiogenic function of CAF in mediating anti-angiogenic drug resistance in ovarian cancers.

A tumor is known to be one of the sources of CAFs in many solid tumors [27,40]. Ovarian stromal cells have similar morphological features to fibroblasts [50]. In ovarian tumors, CAFs have been hypothesized to originate from local fibroblasts, and a histopathological study by Fujisawa et al. demonstrated that local stromal cells are the major source of fibroblasts in ovarian cancers [51]. We tested CAF markers’ (positive and negative) expression pattern(s), including SMA, PD-L1, PD-L2, PD-1, CD31, and S100A4, TE-7, in the resected tumor samples of patients using ICC (Figure 2A–F) to evaluate their expression pattern in the tumor micro-environmental mesenchyme of the resected tumor samples obtained from the same patient whose tissue was used for the culture of CAF. As expected, we observed tumor mesenchyme highly positive for SMA, followed by positivity for S100A4 and TE-7. We standardized a method for the culture of resected tumor-derived CAFs in ovarian cancers to characterize and establish the role of ovarian CAFs in developing resistance to anti-angiogenic lenvatinib using primary cells from patient samples. Recently, the isolation of normal fibroblasts and CAFs from omental tissue using a combination of mechanical dissociation and enzymatic digestion has been reported [52]. We, on the contrary, used resected tumor tissue and the tumor-adjacent normal tissue for the culture in the most life-like way without mechanical dissociation and enzymatic digestion. The primary culture in several passages was tested for the expression of CAF markers before and after freezing (Figure 5).

Activated CAFs are known to express various positive markers, including SMA, FAP-A, and platelet-derived growth factor receptor-A [53]. PDGFR and TGFB signaling are also reported to be associated with the activation of CAFs [54,55]. A high-resolution dissection of the entire tumor ecosystem through single-cell RNA-sequencing analysis of 15 ovarian tumors has been reported by Hornburg et al. [56]. They defined the tumor cells as EpCAM+ CD45-, the stromal cells as EpCAM- CD45-, and the immune cells as EpCAM-CD45+. Their UMAP (uniform manifold approximation and projection) showed the bulk of the cells in the stromal compartment as fibroblast and endothelial cells in ovarian cancers. In line with our results, UMAPs of fibroblasts had a fraction of TGFB-positive CAFs. A single-cell landscape of high-grade serous ovarian cancer demonstrated the transcriptomes of Patient-Derived CAFs [57]. So far, four subtypes of CAF populations (CAF-S1 to CAF-S4) have been identified by analyzing CAF markers in ovarian cancers [41]. Among them, CAF-S1 (FAPhigh/CD29med-high/SMAhigh) and CAF-S4 (FAP−/CD29high/SMAhigh) are associated with cancer progression and metastasis, resembling their activated state as observed from the fact that the mesenchymal high-grade serous ovarian cancer subtype, exhibited high concentrations of CAF-S1 fibroblasts and are associated with poor patient survival [41]. Studies have also shown the existence of distinct subsets of CAF-S1, including myofibroblasts CAFs (myCAFs) with high expression of SMA, as they are located adjacent to cancer cells and produce dense matrices to support tumorigenesis. In line with their study, we observed that CAFs from the tumor cell-dense areas of tumor were highly positive for SMA as tested by qRT-PCR, flow cytometry, Western blot, and ICC (Figure 3, Figure 4 and Figure 5). Hence, we selected these SMA-positive CAFs to test resistance to the anti-angiogenic drug. Interestingly, in the patient with stage IA adult granulosa cell tumor, we observed low expression of FAP-A mRNA parallel to 0.10% expression of FAP-A protein by flow cytometry, which was in contrast to the patient with grade 1, stage IVA low-grade appendiceal mucinous neoplasm whose CAFs exhibited high expression of FAP-A mRNA (Figure 3, Figure 4 and Figure 5) indicating their subtypes as CAF-S4 (FAP-/SMAhigh) and CAF-S1 (FAPhigh/SMAhigh), respectively.

The induction of CAFs in a progressing tumor is contributed by tumor cells which in turn supports tumorigenesis, progressive disease, and drug resistance in response to treatment [58]. CAFs are known to induce angiogenesis directly and indirectly through VEGFA, PDGFC, FGF2, CXCL12, osteopontin, CSF3 secretion, ECM production, and recruitment of myeloid cells [59]. In ovarian cancers specifically, CAFs can induce angiogenesis through the secretion of IL-6, COX-2, and CXCL1 [60], while ovarian cancer cells are reported to induce CAFs to secrete CXCL12, IL-6, and VEGF-A expression via HOXA9, leading to angiogenesis [61]. Ovarian CAFs also induce angiogenesis by secreting VEGF-C via induction by Sonic Hedgehog (SHH) from ovarian cancer cells [62]. The above studies provide a direct role of the "CAFs-tumor cell nexus" in favor of angiogenesis in ovarian cancers. Since we tested the pro-angiogenic effect and anti-angiogenic drug resistance function of CAF, we sought to test the vascularity of the tumor, determining the CD31 positivity of the resected tumor sample. The tumor sample was found to be highly vascular (Figure 2G). Thus, we demonstrate that the particular resected sample tumor from which we used to culture CAF to test the anti-angiogenic resistance, expressed angiogenesis markers, CD31, as well as CAF markers (as mentioned above).

Tumor vessels are known to be distinct from endothelial cell-lined vessels because tumor cells integrate into the endothelium or even mimic and replace it in vasculogenic mimicry vessels [22,27,63]. Cancer cells within a progressive tumor are known to shape their TME via inducing resident fibroblasts and endothelial cells to differentiate into activated CAFs, which in turn produce a qualitatively and quantitatively different ECM. Tumor cells and CAF-secreted vascular endothelial growth factor (VEGF) cause ECs to sprout from pre-existing blood vessels associated with tumor-induced angiogenesis [63]. CAFs are known to secret high levels of ECM proteins, such as fibronectin, one of the most abundant ECM proteins, along with type I and type II collagen, and express oncofetal isoforms of fibronectin (see [64]). in the density and composition of ECM occur in progressing tumors [65]. Thus, CAFs contribute to ECM remodeling, stem features, angiogenesis, immunosuppression, and vasculogenic mimicry, as reported in hepatocellular carcinoma [66]. We observed a significant difference in the formation of CAF clusters between simultaneous co-culture and sequential co-culture at 4 h (Figure 7A,B and Figure 9A,B). The reason for the CAF cluster formation and the difference between the types of cultures will be interesting to study, especially in the context of autocrine and paracrine functions of CAF via secretomes and exosomal vesicles, which is beyond the scope of the present study. In addition to juxtacrine cell contacts, chemokines, and other cytokines [63], CAF-TME-tumor cell crosstalk via secreted exosomes [67] has been known to be effective for the progression of a disease. It is being included in updating therapeutic targeting strategies in different cancers, including pancreatic ductal adenocarcinoma (PDAC) [68]. In fact, it has been postulated that CAFs function as the modulators of crosstalk within ovarian TME involving ECM, angiogenesis, chemoresistance, and invasion in the context of the potential of targeting CAFs as a possible therapeutic approach in ovarian cancers [40]. To find if CAF-derived ECM is enough to initiate cord formation in HUVEC, we tested the cord formation without matrigel. CAFs were placed “on-plate” in a sequential format, and HUVEC cells were plated on CAFs (Figure 10). Thus, our result directly implied for the first time that CAF can replace the need for matrigel, the artificial ECM, inferring that CAF-secreted ECM can be a strong contender for HUVEC cord formation (Figure 10). However, the characterization of the secreted ECM needs to be accounted in the future study. 

Targeting “fibroblast addiction” in primary and metastatic tumors has been long recognized as a novel therapeutic target for drug discovery [69]. With the recent literature support, therapeutic targeting of the TME has been visited recently in highly vascularized solid tumors [70]. CAFs have been shown to increase tumor angiogenesis via pro-angiogenic factors in colon cancers [30]. Anti-angiogenic drugs and CAF-directed normalization of TME and the ECM have been emphasized among several advanced TME-directed therapies, which have either been clinically approved or are currently being evaluated in trials [71,72]. Endothelial and nonendothelial sources of PDGF-B are shown to regulate pericyte recruitment and influence vascular pattern formation in tumors [73]. Interestingly, a PDGF-mediated mesenchymal transformation has been reported to render endothelial resistance (see Figure 3A) to anti-VEGF treatment indicating an endothelial plasticity-mediated mechanism for the control of anti-angiogenic therapy resistance [74]. In ovarian cancers specifically, several reviews postulated the role of CAFs in angiogenesis, lymphangiogenesis, and the development of drug resistance to linking CAFs to the metastatic progression of the disease, searching for a CAF-directed therapeutic strategy employing CAF-associated inhibitors [41,53,75,76,77]. Furthermore, CAFs are known to regulate endothelial adhesion protein lipoma-preferred partner in promoting chemoresistance, highlighting the importance of CAF-endothelial cell crosstalk signaling in ovarian cancers [78]. VEGF secreted by CAFs has been reported to promote angiogenesis in a bevacizumab-resistant manner in oral squamous cell carcinomas [79]. CAFs have been reported to secrete COL1A1 and facilitate the metastasis of ovarian cancer [80], and an oncogenic role of extracellular vesicles-encapsulated leukocyte protease inhibitor secreted by CAFs has been demonstrated in ovarian tumor progression [81]. Hence, “re-education” therapies consisting of switching tumor-supportive stromal cells into tumor-suppressive ones are beginning to gain momentum in the therapeutic management of TME [82]. Our data provide a novel testing platform for the delineation of the specific modalities for CAF-directed crosstalk for angiogenesis in ovarian cancers.

Lenvatinib, an oral multi-targeted TKI of VEGFR1-3, FGFR, PDGFR-β, RET, and KIT, is the representative TKI clinically targeted in ovarian cancers and has been approved by the FDA in combination with pembrolizumab for microsatellite stable recurrent endometrial cancer in 2019 [83] with a number of ongoing clinical trials (*NCT03797326*, *NCT04781088*, *NCT04519151*, *NCT02788708*, and *NCT05114421*) [20]. Yet, escape from anti-angiogenic therapy has been clinically encountered, limiting the anti-angiogenic therapy and indicating a vascular remodeling occurring in tumors that presumably recur during chronic suppression of angiogenesis [84]. In this context, nonendothelial sources of PDGF-B regulating pericyte recruitment and influencing vascular pattern formation in tumors have been reported [73]. The assessment of the local imbalance of endogenous angio-stimulators and angio-inhibitors that promotes tumor-associated leaky vascularization has been shown to be prognosticators of ovarian cancer outcome and may also be regarded as surrogates of ovarian cancer tumor burden and/or ascites formation. Hence, the process of angiogenesis has been targeted for therapeutic development, an unmet need in the context of ovarian cancers with potency to offer an essential direction for managing patient outcomes and treatment [85].

Using a novel HyCC model, we identified that Patient-Derived ovarian CAFs could initiate an immediate angiogenic response in HUVEC cells and specifically resist the effect of the anti-angiogenic drug lenvatinib. Our HyCC model of Patient-Derived ex vivo primary CAFs and HUVEC endothelial cells proved that ovarian CAFs impart resistance to lenvatinib. Here, our data provide the first direct experimental evidence to explain the mechanistic role of ovarian CAF in the development of resistance to anti-angiogenic drugs. The ex vivo HyCC model can potentially evaluate the crosstalk of Patient-Derived endothelium towards developing resistance to anti-angiogenic drugs in ovarian cancers. Our data present a unique experimental tool for personalized testing of anti-angiogenesis drugs in predicting the development of future resistance to anti-angiogenesis drugs, within 3 months of surgery, well before it is clinically encountered in patients. The data in support of CAF-driven resistance to anti-angiogenic drugs are still coming, and it has only just begun.

## Data Availability

We have not used any publicly available data. All data presented in the MS is obtained from the patient samples following informed consent from the patient with proper IRB approval.
